# Identification of a Prognosis-Related Risk Signature for Bladder Cancer to Predict Survival and Immune Landscapes

**DOI:** 10.1155/2021/3236384

**Published:** 2021-10-18

**Authors:** Linhui Wang, Yutao Wang, Jianfeng Wang, Luanfeng Li, Jianbin Bi

**Affiliations:** ^1^Department of Urology, China Medical University, The First Hospital of China Medical University, Shenyang, Liaoning, China; ^2^Department of Pharmacology, China Medical University, School of Pharmacy of China Medical University, Shenyang, Liaoning, China

## Abstract

**Background:**

Bladder cancer is the tenth most common cancer worldwide. Valuable biomarkers in the field of diagnostic bladder cancer are urgently required.

**Method:**

Here, the gene expression matrix and clinical data were obtained from The Cancer Genome Atlas (TCGA), GSE13507, GSE32894, and Mariathasan et al. Five prognostic genes were identified by the univariate, robust, and multivariate Cox's regression and were used to develop a prognosis-related model. The Kaplan–Meier survival curves and receiver operating characteristics were used to evaluate the model's effectiveness. The potential biological functions of the selected genes were analyzed using CIBERSORT and ESTIMATE algorithms. Cancer Therapeutics Response Portal (CTRP) and PRISM datasets were used to identify drugs with high sensitivity. Subsequently, using the bladder cancer (BLCA) cell lines, the role of TNFRSF14 was determined by Western blotting, cell proliferation assay, and 5-ethynyl-20-deoxyuridine assay.

**Results:**

GSDMB, CLEC2D, APOL2, TNFRSF14, and GBP2 were selected as prognostic genes in bladder cancer patients. The model's irreplaceable reliability was validated by the training and validation cohorts. CD8+ T cells were highly infiltrated in the high-TNFRSF14-expression group, and M2 macrophages were the opposite. Higher expression of TNFRSF14 was associated with higher expression levels of LCK, interferon, MHC-I, and MHC-II, while risk score was the opposite. Many compounds with higher sensitivity for treating bladder cancer patients in the low-TNFRSF14-expression group were identified, with obatoclax being a potential drug most likely to treat patients in the low-TNFRSF14-expression group. Finally, the proliferation of BLCA cell lines was increased in the TNFRSF14-reduced group, and the differential expression was identified. TNFRSF14 plays a role in bladder cancer progression through the Wnt/*β*-catenin-dependent pathway. TNFRSF14 is a potential protective biomarker involved in cell proliferation in BLCA.

**Conclusion:**

We conducted a study to establish a 5-gene score model, providing reliable prediction for the outcome of bladder cancer patients and therapeutic drugs to individualize therapy. Our findings provide a signature that might help determine the optimal treatment for individual patients with bladder cancer.

## 1. Introduction

Bladder cancer (BLCA) is the tenth most common cancer worldwide [1]. It is the second most common cancer in men, affecting 13 men per 100,000 (11%), and it is more common in men than in women [2]. Tobacco smoking is the most common risk factor for BLCA, which increases the risk two- to sixfold [3]. BLCA is a heterogeneous disease divided into two types based on the degree of invasion of the lamina propria [4, 5]. Approximately 75% of patients present with non-muscular-invasive BLCA (NMIBC), while the remaining 25% are diagnosed as muscle-invasive BLCA (MIBC) [6]. The primary treatment option of NMIBC is transurethral resection of the bladder tumor followed by chemoradiation [7]. At present, the typical treatment for MIBC includes radical cystectomy and neoadjuvant chemotherapy [8]. Nevertheless, BLCA has a poor outcome despite improved diagnostic technologies and treatment strategies [9, 10]. Therefore, it is essentially significant to identify meaningful predictive methods with relatively higher accuracy to improve outcomes.

Advances in second-generation high-throughput gene sequencing and construction of genome databases of cancers such as TCGA (https://cancergenome.nih.gov/) and Gene Expression Omnibus (GEO, http://www.ncbi.nlm.nih.gov/geo/) have provided extensive sequencing data to explore gene function, including cancer-associated genes. There are prognostically relevant multicohort validated models for many cancer types, including breast cancer [11], oesophagogastric adenocarcinoma [12], and lung cancer [13]. Prognostic models guide the treatment for BLCA patients and provide optimal treatment decisions, thereby improving outcomes as much as possible. In the present study, we sought to establish a reliable predictive model for patients with BLCA. The degrees of freedom of the prognostic model should be limited when it is established, thereby reducing cost. The partial likelihood of the Cox proportional hazard regression model applied to a robust method helps select genes and construct the best model according to the lowest Akaike information criterion scores (AICs). Robust models have higher clinical significance than other methods of constructing prognostic models, including the multivariate Cox regression.

In this study, based on a predictive model for the outcome of BLCA, we identified several protective factors that improve the outcome. The prognostic score model used in this paper is a robust risk model. This model was then verified in four cohorts and applied for different ages, sexes, and pathological stages to help physicians individualize medical decisions for BLCA patients.

## 2. Methods

### 2.1. Data Source

The gene expression matrix and clinical data were obtained from TCGA. Data from 429 BLCA samples were used, including 18 nontumor tissue samples adjacent to the tumor and 411 tumor tissue samples. All BLCA patients included in TCGA were used as the training set. Expression levels of genes were standardized by log_2_(exp + 1). A total of 165 samples with overall survival time, state, clinical stage, sex, and age were obtained from GSE13507 [14] on the GPL6102 Illumina human-6 v2.0 expression bead chip, in addition to this cohort with GSE32894 [15] on the GPL6947 Illumina HumanHT-12 V3.0 expression bead chip from GEO. We also used the cohort from Mariathasan et al. [16]. Expression profile data of human cancer cell lines (CCLs) were obtained from the Broad Institute Cancer Cell Line Encyclopedia project (https://portals.broadinstitute.org/ccle/) [17]. Drug sensitivity data were available in CTRP (v.2.0, released October 2015, https://portals.broadinstitute.org/ctrp), including 481 compounds with over 835 CCLs, and the PRISM Repurposing dataset (19Q4, released December 2019, https://depmap.org/portal/prism/), including 1448 compounds with over 482 CCLs.

### 2.2. The Univariate Cox Regression

We selected differentially expressed genes with median and variability of expression levels higher than 20 percent of all genes. Then, the degree of correlation between gene expression and overall survival was assessed in the training set. We used the “survival” package [18] in R software for the univariate Cox regression analysis of the degree of correlation between gene expression levels and overall survival. Genes associated with the outcome with *p* < 0.05 were selected using “Survdiff” commands in R language, and GraphPad Prism 8.0 was used to draw the Kaplan–Meier survival curves and receiver operating characteristic (ROC) curves of 1, 3, and 5 years to assess their correlations with outcome [19]. Expression of independent prognostic factors was measured at the transcriptional and translational levels [20].

### 2.3. Function Analysis

Gene lists and proteomic studies from high-throughput sequencing were biologically interpreted using the Database for Annotation, Visualization, and Integrated Discovery (DAVID, v6.8), a function enrichment tool, to determine the possible biological functions of the screened genes [21]. DAVID was used to perform the KEGG pathway [22] and enrichment analysis for Gene Ontology [23], and the “ggplot2” package in R was used to generate histograms [24]. Results with *p* < 0.05 are considered significant. Gene set enrichment analysis was used to determine different pathways enriched in the high- and low-TNFRSF14-expression group.

### 2.4. Robust Feature Selection

To create the most stable prognostic model and minimize the degrees of freedom to reduce costs, the principle of robust likelihood-based survival analysis and AICs was applied to screen the genes selected by univariate regression analysis according to the following parameters: iteration times = 100 and max concern genes = 20 [25, 26]. The Kaplan–Meier survival curves and ROC curves [27] were used to represent the meaningful prognostic value of these selected prognostic genes, which were significant (*p* < 0.05) according to the log-rank test [28].

### 2.5. Robust Model Generation

A prognostic model was constructed with the multivariate Cox regression analysis using the five selected robust outcome-related genes. Hazard ratios were used to determine whether a gene is a cancer-promoting (ratio < 1) or cancer-inhibiting factor (ratio > 1). The risk score for each patient was calculated, and the amounts of the five prognostic factors were determined using the “pheatmap” package. The effects were evaluated by drawing the Kaplan–Meier and ROC curves in the training set, test sets, and multiple subgroups.

### 2.6. Immune Environment Evaluation

The Estimation of Stromal and Immune cells in Malignant Tumor tissues using Expression data (ESTIMATE) is an algorithm used to calculate the fractions of stromal and immune cells based on gene expression levels [29]. We calculated the immune score, stromal score, and tumor purity for each BLCA patient using the ESTIMATE method. CIBERSORT is a tool that can calculate the proportion of cells in a tissue sample gene expression profile. LM22 is a gene matrix from the CIBERSORT website portal (https://cibersort.stanford.edu/) used to calculate expression levels of 22 immune cell subtypes [30]. Expression data from LM22 and ESTIMATE were used to analyze expression differences among risk groups. The correlations between expression of prognostic genes and tumor purity, ESTIMATE score, immune score, various inflammatory factors, and others were calculated in R language using the “heat map” package.

### 2.7. Statistical Analysis

All statistical analyses were performed using the R package downloaded from CRAN (https://cran.r-project.org) or BioConductor (http://www.bioconductor.org) in the R language (R x64 4.0.3). The Wilcoxon test was used to compare differences between two groups, and the Kruskal–Wallis test was used to compare differences between multiple groups. If *p* < 0.05, differences were considered statistically significant; if *p* < 0.01, there was a highly significant difference between the groups.

### 2.8. Cell Culture and Transfection

The BLCA cell lines T24 and UMUC3 were purchased from the Chinese Academy of Sciences Cell Bank (China). T24 and UMUC3 were cultured in RPMI 1640 and DMEM (high-glucose) medium (Hyclone) containing 10% fetal bovine serum, respectively. Both cell lines were cultured at 37°C with 5% CO_2_. Small interfering RNAs (siRNA) targeting TNFRSF14 to reduce its expression were purchased from JTSBIO Co. (China). The si-TNFRSF14 (H) -539 (#1) sequences were as follows: sense, CCUACAUUGCCCACCUCAATT; anti-sense, UUGAGGUGGGCAAUGUAGGTT. The si-TNFRSF14 (H) -941 (#2) sequences were as follows: sense, GCUCCACAGUUGGCCUAAUTT; anti-sense, AUUAGGCCAACUGUGGAGCTT.

### 2.9. Total RNA Extraction and Quantitative Real-Time PCR (qRT-PCR)

Total RNA was extracted using RNAiso Plus (Takara Biotechnology, Dalian, China). After the manufacturer's instruction, total RNA was reverse transcribed into cDNA with PrimeScript RT Master Mix (Takara, Dalian). SYBR® Premix Ex Taq™ Kit (Takara, Dalian) was used to perform qRT-PCR. The Thermal Cycler Dice™ Real-Time TP800 system (Takara, Kyoto) was used to perform all analyses. GAPDH was used as an internal reference, and the relative expression level of a gene was calculated by the ΔΔCT method. The primer sequences are listed as follows: TNFRSF14 (forward: GTGCAGTCCAGGTTATCGTGT; reverse: CACTTGCTTAGGCCATTGAGG) and GAPDH (forward: GGAGCGAGATCCCTCCAAAAT; reverse: GGCTGTTGTCATACTTCTCATGG).

### 2.10. Western Blotting (WB)

Cells were lysed in radioimmunoprecipitation assay (RIPA) buffer, protein was taken, and the concentrations were measured using a bicinchoninic acid assay kit. The proteins were separated using 10% sodium dodecyl sulfate-polyacrylamide gel electrophoresis (SDS-PAGE), and the proteins were transferred to polyvinylidene fluoride membranes. Subsequently, the membranes were blocked using Tris-buffered saline with 1% Tween 20 (TBS-T) with 5% nonfat milk at 37°C for 1 h. After adding primary antibodies for *β*-catenin and cyclin D1, the membranes were incubated at 4°C overnight. After washing three times with TBS-T, the membranes were incubated with a secondary antibody for 1 hour at 37°C and then washed again. The enhanced chemiluminescence method was used to observe the protein expression, and ImageJ software was used to calculate the protein expression value.

### 2.11. Cell Proliferation Assay

T24- and UMUC3-treated cells were seeded in 96-well plates, and the Cell Counting Kit-8 (CCK-8) assay reagent (Dojindo Molecular Technologies, Inc.) was added to each well following the manufacturer's instructions. An absorbance reader (Bio-Rad) was used to measure the absorbance at a wavelength of 450 nm.

### 2.12. Ethynyl-20-Deoxyuridine Assay (EdU)

The proliferation ability of the cells was detected with the 5-ethynyl-20-deoxyuridine (EdU) assay kit (RiboBio, Guangzhou, China). A total of 2000 cells were added to each well in a 96-well plate after transfection. After 18 h, 500 *μ*l medium containing 50 *μ*M EdU was added to each well for 3 hours. After aspirating the culture medium, 4% paraformaldehyde was added to each well to fix the cells at room temperature for 15 min. Subsequently, 0.3% Triton X-100 in phosphate-buffered saline was added to each well at room temperature for 15 min, and then the cells were incubated for 30 min with click reaction solution. Finally, Hoechst 33342 diluted in phosphate-buffered saline (1 : 1000) was added to each well at room temperature for 10 min. The proportion of EdU-positive cells was observed with a fluorescence microscope (Olympus Corporation, Japan) and photographed. The percentage of EdU-positive cells was calculated using the ImageJ software (NIH Image, Bethesda, MD, USA).

## 3. Results

### 3.1. Data Source

The expression levels and clinical data of 429 BLCA samples were acquired from TCGA, including 410 tumor samples and 19 nontumor samples. GSE13507 (*n* = 165) and GSE32894 (*n* = 224) were used. These patient samples included RNA expression, overall survival time, survival status, clinical stage, gender, and age. We also used the cohort from Mariathasan et al. (*n* = 195).

### 3.2. The Univariate Cox Regression

**Figure 1:**
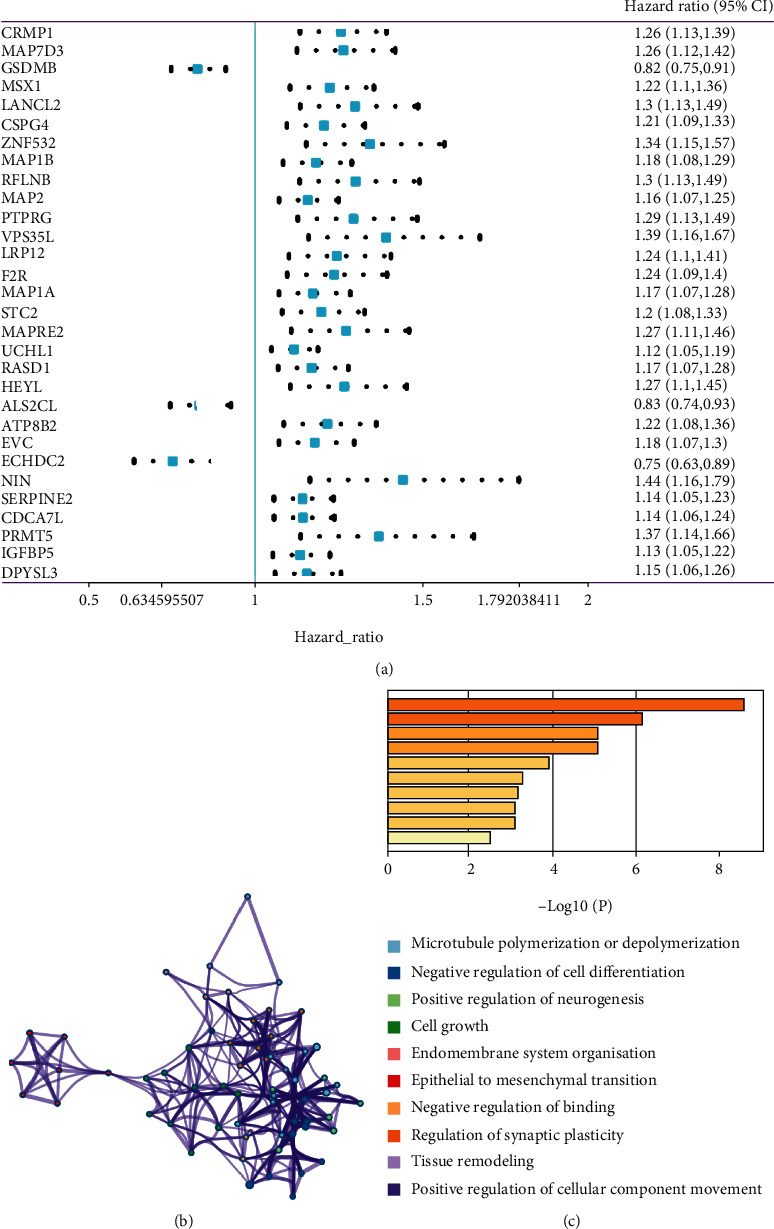
The univariate Cox regression analysis and enrichment analysis. (a) The univariable Cox regression of the top 30 genes for overall survival. (b) The interaction network map of enriched proteins; the same color indicates the same enrichment. (c) The enriched terms decrease from top to bottom by the significance of enrichment.

Using all BLCA samples from TCGA as a training set, a total of 829 outcome-related genes with *p* < 0.05 were identified using the univariate Cox regression analysis. The top 30 genes are shown in Figure 1(a). Hazard ratios were used to identify risk factors or protective factors: HR > 1 suggests that a gene is a risk factor, while genes with HR < 1 are protective factors. Of the top 30 genes, 27 were risk factors, and the remaining genes were protective factors.

### 3.3. Function Analysis

**Figure 2:**
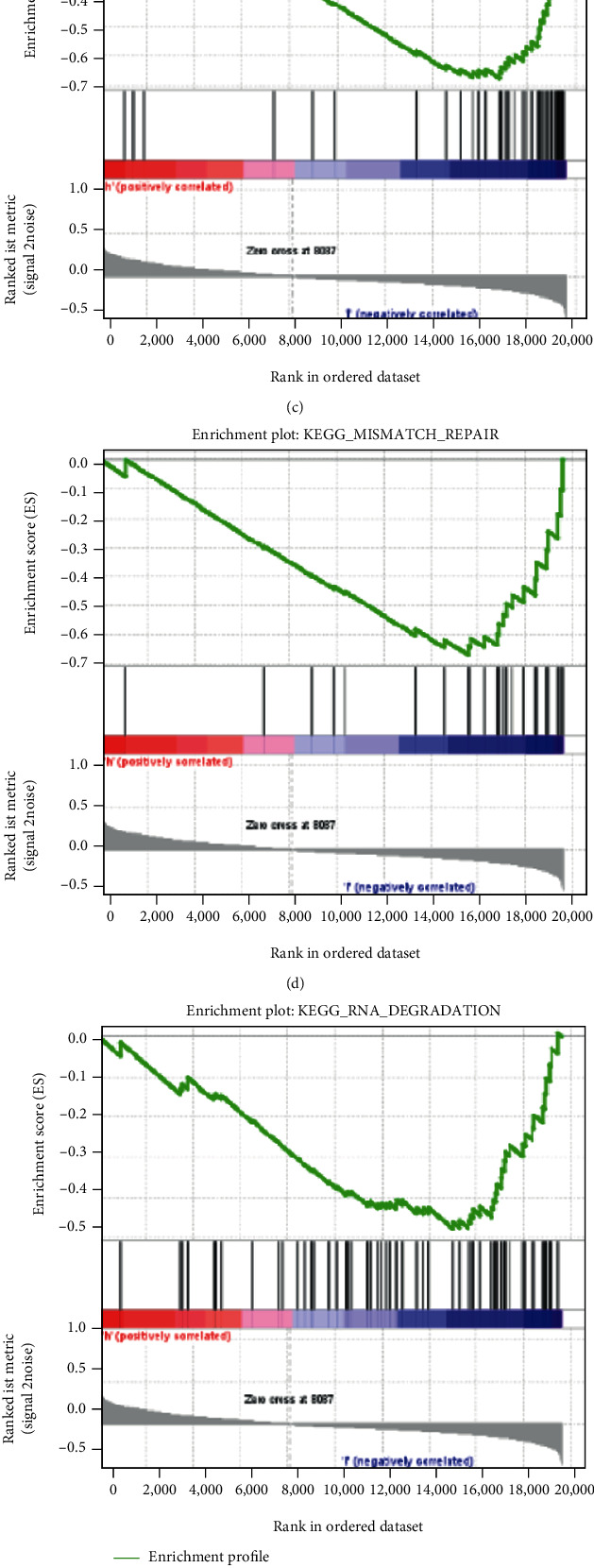
Analysis related to high- and low-TNFRSF14-expression group. (a–e) Pathways with differences. (f–g) Drugs with higher sensitivity in the low-TNFRSF14-expression group.

Pathway and biological process enrichment analyses of these selected genes were performed to explore further their possible biological functions (Figures 1(b) and 1(c)). The enrichment terms from top to bottom are arranged from high to low according to the significance of the enrichment. Analysis of pathways enriched in high- and low-TNFRSF14-expression groups was performed to explore the pathways involved in TNFRSF14 (Figures 2(a)–2(e)).

### 3.4. Robust Feature Selection

In the first step, the training set (*N* = 429 samples) was randomly divided into a subtraining set with *N*∗(1 − *p*) samples and a subvalidation set with *N*∗*p* samples (*p* = (1/3)). A gene was fitted to the subtraining set of samples, and the parameter estimate was obtained. Then, log-likelihood with the parameter estimate and the subvalidation set were evaluated. This evaluation was performed for each gene.

In the second step, the procedure was repeated over 100 times. In this manner, 100 times log-likelihood for each gene were obtained. The best genes with the smallest mean negative log-likelihood (or the largest mean log-likelihood) were selected. The best genes were the ones most closely associated with survival selected by the robust likelihood-based approach.

In the third step, Gene A was regarded as the best-selected gene in the previous step. We found the next best gene (B) by repeating the previous two steps.

In the fourth step, this selection was continued until there were no more samples, resulting in a series of *K* models *M*1 = *A*, *M*2 = *A* + *B*, and *MK* = *A* + *B* + ⋯+*K*.

**Figure 3:**
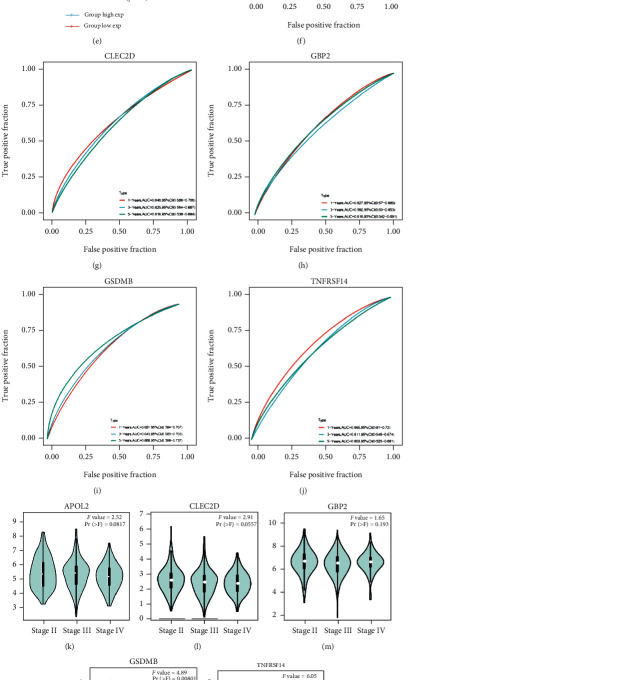
Effects of the single factor on patient outcome in TCGA cohort. (a–e) The Kaplan–Meier survival curves for patients with BLCA in TCGA, stratified according to the expression levels of APOL2, CLEC2D, GBP2, GSDMB, and TNFRSF14 (high vs. low); comparisons of the median survival time in both groups with log-rank tests (*p* = 0.00051, *p* < 0.0001, *p* = 0.014, *p* = 0.00027, and *p* < 0.0001, respectively). (f–j) ROC curve analysis of the prognostic accuracy of APOL2, CLEC2D, GBP2, GSDMB, and TNFRSF14 in TCGA. (k–o) Comparisons of the expression levels of various genes in the robust model in TCGA for different stages.

In the final step, the best genes that were marked as (∗) were selected in the model with the smallest AIC, which was considered the most stable and sound model with the least number of degrees of freedom (i.e., GBP2, TNFRSF14, APOL2, CLEC2D, and GSDMB), as shown in Table 1. The Kaplan–Meier analysis and ROC curves indicated that all five factors were protective (Figures 3(a)–3(j)). The correlation between the expression of the five genes and the clinical stage is shown in Figures 3(k)–3(o). Afterward, the independent prognostic value of the five selected genes was verified in three external GEO cohorts (Supplementary Figures 1–3).

### 3.5. Robust Model Generation

Using the multivariable Cox regression analysis, a risk score model was established with the five selected robust genes to calculate the correlation between the comprehensive expression levels of the five genes and patients' outcomes (Table 2). The risk score formula was as follows: −0.113∗GSDMB − 0.167∗CLEC2D − 0.029∗APOL2 − 0.088∗TNFRSF14 − 0.176∗GBP2.

**Figure 4:**
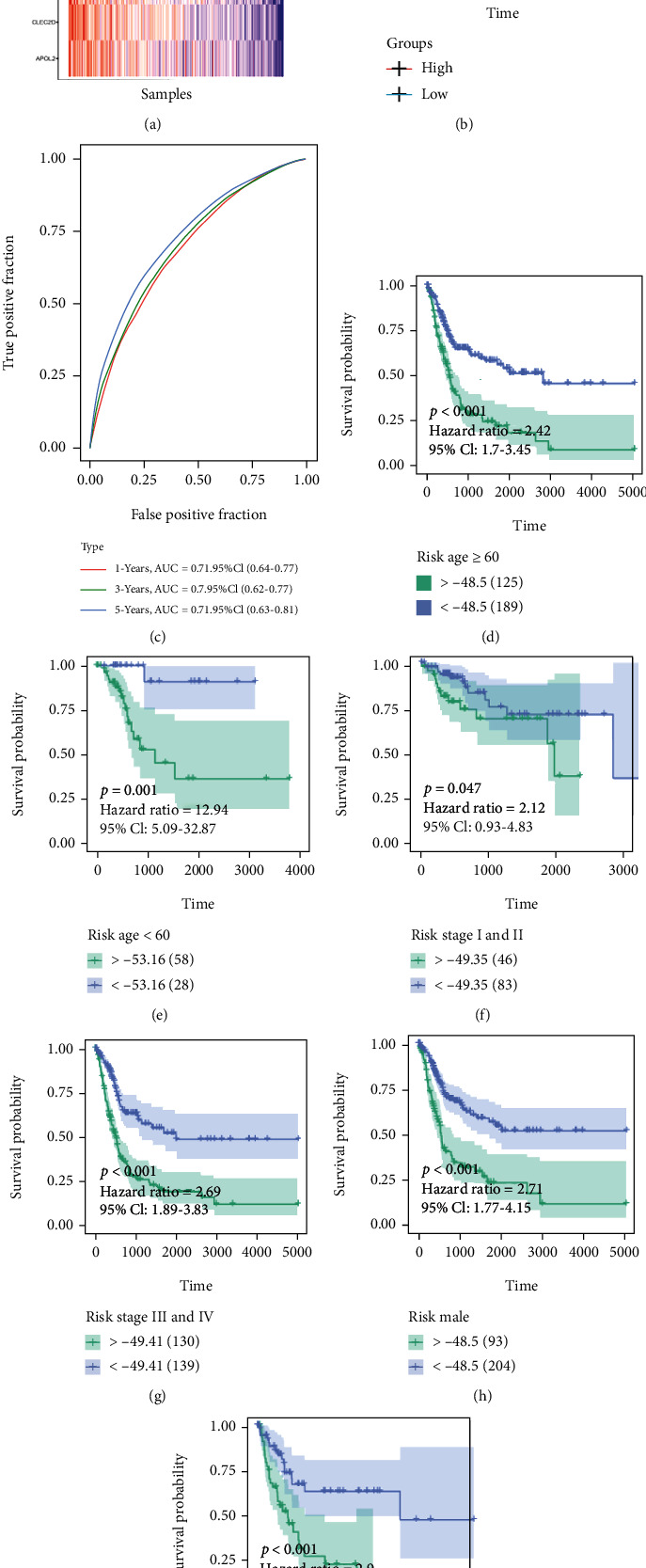
Effects of the robust model on patient outcome in TCGA cohort. (a) The risk score analysis is from top to bottom: patient's risk distribution, gene expression profile, and survival status map. (b) The Kaplan–Meier survival curves for patients with bladder cancer in TCGA, stratified according to risk scores (high vs. low); comparisons of the median survival time in both groups with log-rank tests (*p* < 0.0001). (c) ROC curve analysis of the prognostic accuracy of the model in TCGA. (d–i) The subgroups' Kaplan–Meier's analysis of risk score.

The risk score formula was applied to calculate the risk value of each sample in the test set and the obtained value of the risk score. The survival status and the expression levels of the five selected genes for each sample are shown in Figure 4(a). The Kaplan–Meier analysis revealed that outcomes in the high-risk group were significantly worse than those of patients in the low-risk group with *p* < 0.0001 (Figure 4(b), HR = 2.72). An ROC curve was drawn to determine the constructed model's reliability, and the relationship between risk scores and disease types was queried. Area under the curve (AUC) values at various periods are shown in Figure 4(c) (1/3/5 year = 0.71/0.70/0.71).

### 3.6. Subgroup Analysis

The samples in the training set were divided into subgroups to assess the reliability of the constructed model, including age, clinical stage, and sex. The survival curves indicated that the model was significant in each subgroup (*p* < 0.05) (Figures 4(d)–4(i)). Meanwhile, the prognostic significance of the above five-gene risk signature in the different subgroups of GSE13507 is significant (Supplementary Figure 4).

### 3.7. Signature Verification

**Figure 5:**
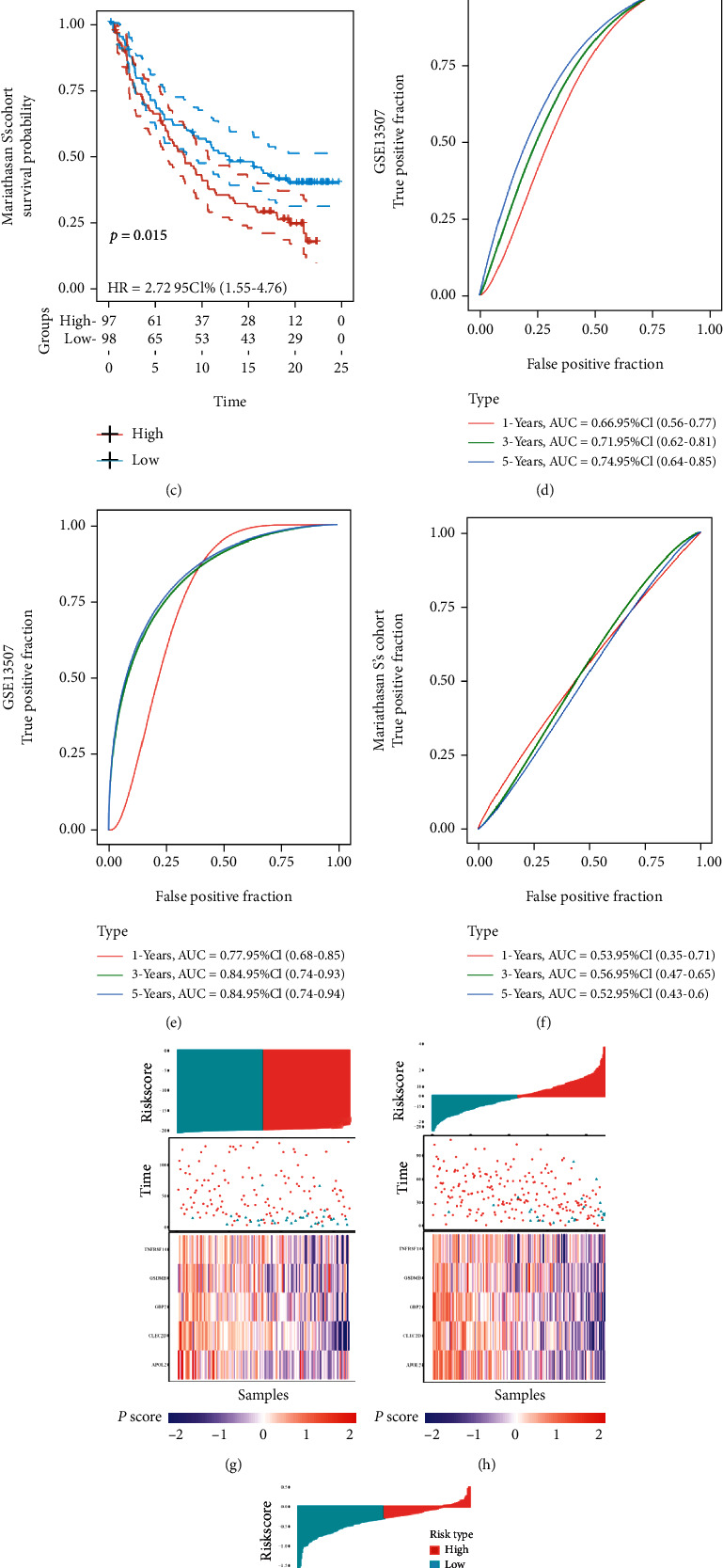
Effects of the robust model on patient outcome in external cohorts. (a–c) The Kaplan–Meier survival curves for patients with BLCA in GSE13507, GSE32894, and Mariathasan S's cohort, stratified according to risk scores (high vs. low); comparisons of the median survival time in both groups with log-rank tests (*p* = 0.011, *p* < 0.0001, and *p* = 0.015, respectively). (d–f) Receiver operating characteristic curve analysis of the prognostic accuracy of the model. (g–i) The risk score analysis is from top to bottom: patient's risk distribution, gene expression profile, and survival status map.

Three external cohorts (GSE13507, GSE32894, and the Mariathasan cohort) were used for validation to validate the model's reliability further. The survival curves indicated that the model was significant in all three cohorts, i.e., patients in the high-risk group had worse outcomes than those in the low-risk group. *p* values in the three cohorts were 0.011, <0.0001, and 0.015, respectively (Figures 5(a)–5(c)). The ROC curves of the four cohorts are shown in Figures 5(d)–5(f). The 1-, 3-, and 5-year AUCs of the three cohorts were 0.66/0.71/0.74, 0.77/0.84/0.84, and 0.53/0.56/0.52, respectively.

The risk score formula was also applied to calculate the risk value of each sample in the four cohorts and the obtained value of the risk score. The survival status and the expression levels of the five selected genes for each sample are shown in Figures 5(g)–5(i).

### 3.8. Immune Environment Evaluation

**Figure 6:**
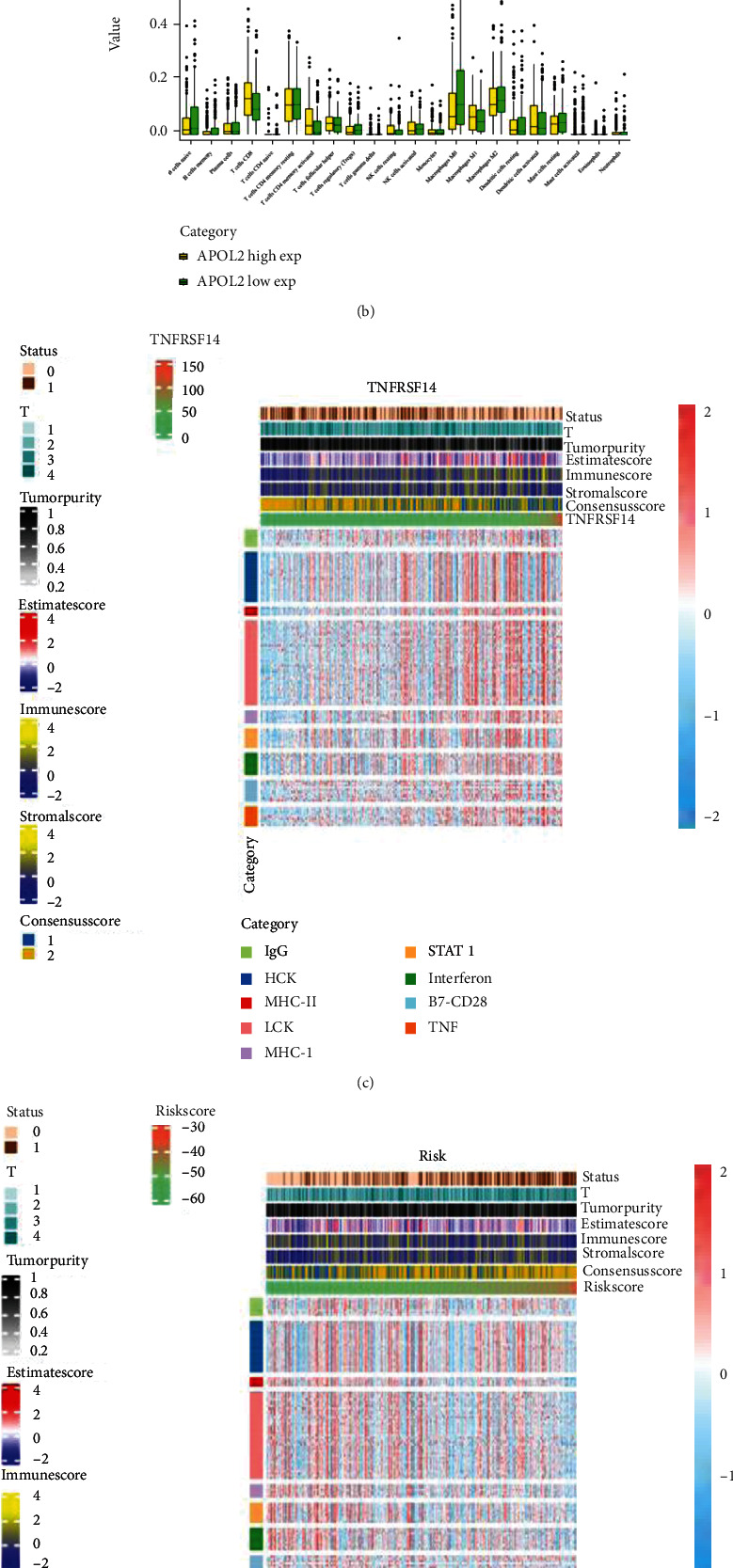
Tumor microenvironment analysis. (a, b) The difference of 22 kinds of immune cells between the high-expression group and the low-expression group according to the expression levels of TNFRSF14 and APOL2 was analyzed and shown by box plots. *p* < 0.05 was marked as ∗; *p* < 0.01 was marked as ∗∗; *p* < 0.001 was marked as ∗∗∗; *p* < 0.0001 was marked as ∗∗∗∗. (c, d) The correlation of the immunoproteasome of TNFRSF14 and risk score to survival status, TumorPurity, and inflammatory and immune responses. These responses were induced by immunoglobulin G (IgG), HCK, major histocompatibility complex class II (MHC-II), lymphocyte-specific kinase (LCK), major histocompatibility complex class I (MHC-I), activator of transcription 1 (STAT1), interferon, B7-CD28, and TNF.

CIBERSORT was used to calculate the expression of 22 immune cells from all BLCA samples in TCGA. All samples were divided into high- and low-TNFRSF14-expression and high- and low-APOL2-expression groups, respectively. The differences in expression levels of 22 immune cells between high- and low-expression groups were displayed in box plots. The results showed that CD8+ T cells were highly infiltrated in the high-TNFRSF14-expression group, and M2 macrophages were less highly expressed in the high-TNFRSF14-expression group. This was also the case in the high- and low-APOL2-expression groups (Figures 6(a) and 6(b)). The heat map showed that higher expression of TNFRSF14 was associated with better survival status; lower tumor stage, tumor purity, ESTIMATE score, immune score, and stromal score; and higher expression levels of various inflammatory factors, including IgG, hemopoietic cell kinase (HCK), MHC-2, and others (Figure 6(c)). An opposite trend was observed for the heat map of the risk score (Figure 6(d)). Immune microenvironment analysis of other factors had been added to Supplementary Figure 5.

### 3.9. Identification of Potential Therapeutic Agents for Low-TNFRSF14-Expression BLCAs

There are gene expression and drug response data from hundreds of cell lines for CCLs in the CTRP and PRISM datasets, which can be used as a test set for drug response. Both datasets had a total of 1770 compounds, and we excluded samples and cell lines with more than 20% NA in hematopoietic and lymphoid tissues. *K*-nearest neighbor (*k*-NN) imputation was used to fill in the missing AUC values. Finally, 354 compounds in the CTRP dataset and 1285 PRISM datasets were used for subsequent analysis. Subsequently, the pRRophetic package was applied to predict the effect of candidate drugs applied to clinical patients, and the corresponding estimated AUC values were obtained. AUC values are negatively related to the sensitivity of the drug.

Two methods were applied for cross-analysis to identify candidate drugs with higher drug sensitivity for patients with low TNFRSF14 expression based on CTRP and PRISM-derived drug response. First, differential analysis of drug response was performed in the high- (top decile) and low-TNFRSF14- (bottom decile) expression groups to identify compounds with lower estimated AUC values in the low-TNFRSF14-expression group (log_2_FC < −0.10). Next, the AUC values and TNFRSF14 expression were subjected to Spearman's correlation analysis to select compounds with positive correlation (Spearman's *r* > 0.20 for CTRP or 0.30 for PRISM). Finally, 12 compounds in CTRP (including BI-2536, GSK461364, KX2-391, leptomycin B, obatoclax, paclitaxel, panobinostat, PI-103, rigosertib, SB-743921, vincristine, and YM-155) and 14 compounds in PRISM (including AC-264613, atorvastatin, combretastatin-A-4, docetaxel, epothilone-b, GZD824, ispinesib, litronesib, mitoxantrone, NVP-AUY922, obatoclax, quizartinib, teniposide, and volasertib) were screened out. All these compounds had lower estimated AUC values in the TNFRSF14 low-expression group and a positive correlation with TNFRSF14 expression (Figures 2(f) and 2(g)).

### 3.10. Knockdown of TNFRSF14 Significantly Inhibited the Wnt Pathway and BLCA Cell Proliferation

**Figure 7:**
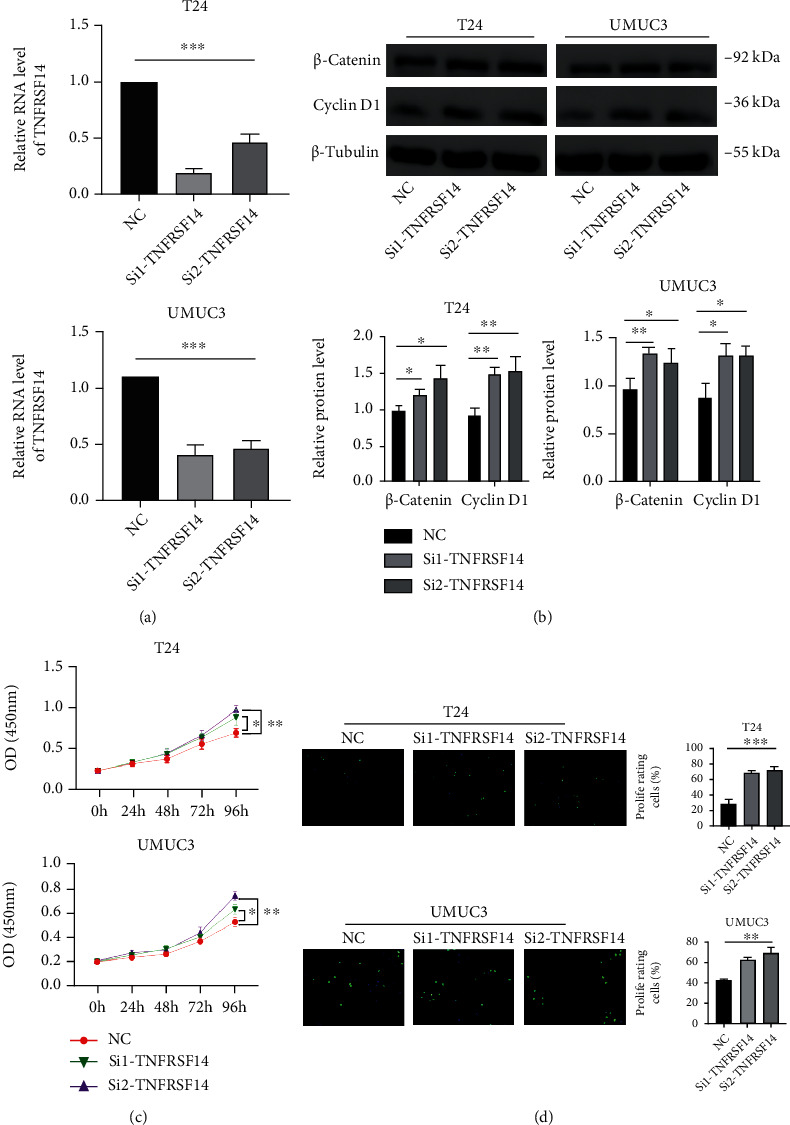
Experiment of TNFRSF14. (a) Relative RNA level of TNFRSF14 in T24 and UMUC3 cells following TNFRSF14 knockdown. GAPDH served as loading control. (b) Relative protein level of *β*-catenin and cyclin D1 in T24 and UMUC3 cells following TNFRSF14 knockdown. *β*-Tubulin served as loading control. (c) The effects of TNFRSF14 on the proliferation of T24 and UMUC3 cells were analyzed by CCK-8 assays. The results are presented as the mean optical density (OD) at 450 nm for triplicate wells. The results are presented as the mean ± SD of three independent experiments (^∗^*p* < 0.05; ^∗∗^*p* < 0.01). (d) EdU incorporation assays were used to determine the effects of TNFRSF14 on T24 and UMUC3 cell proliferation. The ratio of EdU-positive cells (green) per field to the number of Hoechst 33342-positive cells (blue) in the same field was calculated in three random fields.

TNFRSF14 is poorly expressed in BLCA and is associated with a good outcome. In BLCA cell lines, the role of TNFRSF14 was validated by in vitro experiments. The expression of TNFRSF14 in two cell lines, T24 and UMUC3, is knocked down with siRNA-TNFRSF14, and the expression levels of TNFRSF14 after knockdown are shown in Figure 7(a). Protein expression of both *β*-catenin and cyclin D1 were increased after the knockdown of TNFRSF14 (Figure 7(b)). The CCK-8 assay was used to determine the effect of TNFRSF14 on the proliferation ability of T24 and UMUC3 cell lines. TNFRSF14 inhibited the proliferation ability of BLCA cells (Figure 7(c)). The proliferation ability of TNFRSF14 for BLCA cells was then also determined again using the EdU assay, and the result was consistent with the CCK-8 assay (Figure 7(d)).

## 4. Discussion

BLCA is one of the most common urinary tumors, with high incidence, recurrence, and variable outcomes [31–33]. There is no reliable predictive method for predicting prognosis and guiding individualized treatment. In this study, we established a robust prognostic score model for BLCA. All samples were divided into subgroups, including stage, age, and gender, and our model was analyzed in each subgroup. The model was built using RNA-seq data from all BLCA samples in TCGA and was validated using three external test sets. The prognostic score model contains five protective factors (GSDMB, CLEC2D, APOL2, TNFRSF14, and GBP2). The potential biological functions of these five genes were analyzed. We found that CD8+ T cells and M2 macrophages were closely related to TNFRSF14 and APOL2, and the expression of TNFRSF14 and risk score were strongly associated with tumor purity, immune score, various inflammatory factors, and others. These findings hold implications for future clinical and biological research.

With the rapid development of bioinformatics analysis, there have been many studies predicting risk models for BLCA. These models have included too many factors that increase costs and burdens on patients. Furthermore, the reliability of each factor in these models alone was insufficiently strong, suggesting that these factors are not the most appropriate predictive markers.

Cancer stem cells (CSCs) can affect tumor progression, recurrence, metastasis, and resistance to therapy [34, 35], and there are many CSC markers, including OCT4 [36] and CD133 [37]. Chan et al. suggested that CSCs can promote the progression of BLCA [38]. Sedaghat et al. suggested that OCT4/CD133 can be efficiently used for early diagnosis and for determining the prognosis of patients with BLCA, but OCT4 and CD133 cannot be used as independent prognostic factors for BLCA [39]. In the present study, GSDMB, CLEC2D, APOL2, TNFRSF14, and GBP2 in our constructed model can be considered as independent prognostic factors in BLCA.

Pyroptosis is a novel programmed cell death mechanism discovered in recent years [40]. Zhou et al. suggested that the expression of GSDMB promotes pyroptosis in 293T cells; the higher expression of GSDMB is correlated with better outcomes in bladder carcinoma and cutaneous melanoma [41]. The GSDMB protein promotes pyroptosis through its N-domain in HEK293T cells [42]. The expression of GSDMB in BLCA tissues is lower than that in normal tissues, and the high expression of GSDMB is significantly correlated with the good prognosis of BLCA [41].

Inflammation is an essential process in the tumor microenvironment mediated by a mixture of cytokines secreted by tumors and immune cells [43, 44]. In the interaction between the tumor and the immune system, cytokines exert biological functions in the tumor microenvironment by activating immune cells and stimulating inflammation [45]. Inflammatory reactions recognize and eliminate specific antigens from tumor cells (a process called immune monitoring) and prevent cancer [46]. Del Fresno and Sancho suggested that CLEC2D mediates cytokine-driven inflammation, and the CLEC2D pathway can enhance other inflammatory responses and the antitumor process [47]. Mathew et al. suggested that CLEC2D might allow prostate cancer cells to evade the immune system by inhibiting natural killer cells [48]. Tang et al. suggested that CLEC2D can enhance prostate cancer resistance to docetaxel [49].

Apolipoprotein L2 (APOL2) belongs to the L family of lipid-binding proteins [50–52]. The APOL gene family is involved in programmed cell death and initiates apoptosis or autophagic death [53]. Gupta et al. suggested that APOL2 was a proinflammatory gene [54]. APOL2 might be antiapoptotic to inhibit progression and is a prognostic factor for BLCA [50–52].

Tumor necrosis factor receptor superfamily 14 (TNFRSF14), a protein encoded by this gene which is also called HVEM, encodes members of the tumor necrosis factor (TNF) receptor superfamily and activates proinflammatory pathways [55]. TNFRSF14 mediates apoptosis and inhibits tumor cells from undergoing immune escape [56, 57]. TNFRSF14 can inhibit the proliferation of BLCA by promoting apoptosis and is a prognostic marker for BLCA [58].

Guanylate-binding protein 2 (GBP2) is a member of the GBP family. When GBP2 is absent in macrophages, there are blunted immunological responses, suggesting that it plays an essential role in the inflammatory process [59, 60]. The upregulation of GBP2 gene expression is positively correlated with outcomes in cutaneous melanoma [61]. Wang et al. suggested that GBP2 inhibits the proliferation and invasion of colorectal cancer cells [62]. Zhang et al. suggested that GBP2 inhibits breast cancer cell invasion [63]. Godoy et al. found that GBP2 is a protective factor in breast cancer [64]. Messmer-Blust et al. found that upregulation of GBP2 inhibits cell spreading [65]. GBP2 might be a target for improving BCG efficacy and is a prognostic protective factor for BLCA [66].

Measurement of the degree of 22 immune cell infiltration suggested that the expression of TNFRSF14 and APOL2 is strongly correlated with CD8+ T cells and M2 macrophages. Jansen et al. suggested that BLCA patients with a high degree of infiltration of CD8+ T cells had good outcomes and strong responses to immunotherapy [67]. In BLCA, tumor-associated macrophages are associated with the M2 phenotype [68], and M2 macrophages are associated with poor outcomes [69]. Our present findings are consistent with these conclusions.

Enhancement of antitumor activity requires tumor neoantigen-specific CD8+ T cells to inhibit tumors via major histocompatibility complex- (MHC-) I [70] and enhanced immunotherapy by activating CD4+ T cells via MHC-II [71]. The antitumor ability of MHC-II-restricted CD4+ T cells is particularly strong [72]. Upregulation of MHC-II expression enhances antigen presentation and prevents tumor immune escape [73, 74] regarding adaptive immune responses, in which the Src-family tyrosine kinase LCK [75] activates T cell receptors and enhances immune responses [76].

Interferon signaling is involved in the immune surveillance mechanism of tumors [77]. Interferon-*γ*, secreted by CD8+ cytotoxic T lymphocytes, inhibits tumors, an essential part of antitumor immune responses [78]. IFN-*α* expands CD8+ T cells on a large scale and blocks the cycle of cancer cells, thereby improving outcomes [79]. In our study, the expression of TNFRSF14 is positively correlated with the expression levels of MHC-I, MHC-II, LCK, and interferon, while the risk score was the opposite. This result also indicated that the higher expression of TNFRSF14 is correlated with lower tumor purity, while the risk score was the opposite.

The Wnt signaling pathways were divided into canonical pathways, Wnt/*β*-catenin-dependent pathways, and noncanonical Wnt pathways [80]. The proliferation ability of bladder cancer cells can be improved by activating the Wnt pathway [81]. Cyclin D1 is a downstream target of the Wnt/*β*-catenin-dependent pathway [82], and cyclin D1 can also promote the proliferation of BLCA cells [83].

Obatoclax can be used to reduce the resistance of BLCA cells to paclitaxel [84], and paclitaxel (PTX) is a widely used drug in clinical practice and can be used to treat BLCA [85].

Although the five-gene robust model established in this study predicted outcomes in BLCA, there remain some limitations of the present study. Previous investigators provided the patient data. There were also few cohorts for external validation. Furthermore, the reliability of the robust model was only analyzed at the sequencing level and was not clinically validated. Panobinostat (PAN) can increase the sensitivity of muscle-invasive BLCA cells to radiation [86]. Treatment with docetaxel can reduce the size of bladder tumors [87]. Obatoclax was identified in both CTRP and PRISM as a more sensitive compound in the low-TNFRTF14-expression group, so it may be a potential drug most likely to treat patients in the low-TNFRSF14-expression group.

## 5. Conclusion

In conclusion, a reliable risk score model was established to predict outcomes in patients with BLCA and identify the screened genes' possible biological functions. CD8+ T cells and M2 macrophages were related to the expression of the selected genes. Expression levels of MHC-I, MHC-II, LCK, and interferon were associated with the expression of THFRSF14 and the risk score. Tumor purity is correlated with the expression of THFRSF14 and the risk score. Obatoclax may be a potential drug most likely used to treat patients in the low-TNFRSF14-expression group. Nevertheless, further studies are needed to validate the stability of the predictions of the established model and evaluate its practical value in clinical applications.

## Figures and Tables

**Table 1:** The result of robust gene model in TCGA - BLCA.

Seq	Order	Gene	nloglik	AIC	Selected
1	0	0	854.81	1709.62	
1	1	GBP2	841.69	1685.39	^∗^
1	2	TNFRSF14	837.46	1678.91	^∗^
1	3	APOL2	836.23	1678.46	^∗^
1	4	CLEC2D	832.52	1673.03	^∗^
1	5	GSDMB	830.87	1671.74	^∗^
1	6	CXorf38	830.68	1673.36	
1	7	OAS1	830.35	1674.71	
1	8	PSMB10	830.35	1676.7	
1	9	OFD1	830.33	1678.66	
1	10	RNF19A	828.15	1676.3	
1	11	ECHDC2	828.15	1678.3	
1	12	CD96	827.61	1679.23	
1	13	DOCK8	827.29	1680.59	
1	14	CARD8	826.8	1681.59	
1	15	ALPK1	826.8	1683.59	
1	16	TMEM229B	826.54	1685.08	
1	17	ANAPC4	826.52	1687.03	
1	18	PCED1B	825.94	1687.88	
1	19	TRIM38	825.86	1689.72	

Nloglik: negative log-likelihood; AIC: Akaike information criterion score.

**Table 2:** Variables in the equation.

	B	SE	Wald	Df	Sig	Exp(B)	95% CI of Exp(B)
GSDMB	-0.113	0.062	3.265	1	0.071	0.893	0.790-1.010
CLEC2D	-0.167	0.090	3.400	1	0.065	0.846	0.709-1.011
APOL2	-0.029	0.091	0.100	1	0.751	0.972	0.813-1.162
TNFRSF14	-0.088	0.099	0.786	1	0.375	0.916	0.754-1.112
GBP2	-0.176	0.079	5.004	1	0.025	0.838	0.718-0.978

Df: degree of freedom; Sig: significance.

## Data Availability

The TCGA-BLCA dataset used in this study could be obtained from the TCGA database (https://cancergenome.nih.gov/). This study's two GEO datasets (GSE13507, GSE32894) were obtained from the GEO database (https://www.ncbi.nlm.nih.gov/geo/).

## Supplementary Materials

Supplementary Materials Supplementary Figure 1: effects of the single factor on patient outcome in GSE13507. (A–E) The Kaplan–Meier survival curves for patients with bladder cancer in GSE13507, stratified according to the expression levels of APOL2, CLEC2D, GBP2, GSDMB, and TNFRSF14 (high vs. low); comparisons of the median survival time in both groups with log-rank tests (*p* = 0.049, *p* = 0.0056, *p* = 0.0018, *p* = 0.049, and *p* = 0.027, respectively). (F–J) ROC curve analysis of the prognostic accuracy of APOL2, CLEC2D, GBP2, GSDMB, and TNFRSF14 in GSE13507. Supplementary Figure 2: effects of the single factor on patient outcome in GSE32894. (A–E) The Kaplan–Meier survival curves for patients with BLCA in GSE32894, stratified according to the expression levels of APOL2, CLEC2D, GBP2, GSDMB, and TNFRSF14 (high vs. low); comparisons of the median survival time in both groups with log-rank tests (*p* < 0.0001, *p* = 0.00021, *p* = 0.00049, *p* = 0.00053, and *p* = 0.037, respectively). (F–J) ROC curve analysis of the prognostic accuracy of APOL2, CLEC2D, GBP2, GSDMB, and TNFRSF14 in GSE32894. Supplementary Figure 3: effects of the single factor on patient outcome in Mariathasan S's cohort. (A–E) The Kaplan–Meier survival curves for patients with BLCA in Mariathasan S's cohort, stratified according to the expression levels of APOL2, CLEC2D, GBP2, GSDMB, and TNFRSF14 (high vs. low); comparisons of the median survival time in both groups with log-rank tests (*p* = 0.018, *p* = 0.0018, *p* = 0.03, *p* = 0.0064, and *p* = 0.22, respectively). (F–J) ROC curve analysis of the prognostic accuracy of APOL2, CLEC2D, GBP2, GSDMB, and TNFRSF14 in Mariathasan S's cohort. Supplementary Figure 4: effects of the robust model on patient outcome in GSE13507. (A–F) The subgroups' Kaplan–Meier analysis of risk score. Supplementary Figure 5: tumor microenvironment analysis. (A–C) The difference of 22 kinds of immune cells between the high-expression group and the low-expression group according to the expression levels of CLEC2D and GBP2 and the low-risk and high-risk groups were analyzed and shown by box plots. *p* < 0.05 was marked as ∗; *p* < 0.01 was marked as ∗∗; *p* < 0.001 was marked as ∗∗∗; *p* < 0.0001 was marked as ∗∗∗∗. (D–F) The correlation of the immunoproteasomes of CLEC2D, GBP2, and APOL2 to survival status, TumorPurity, and inflammatory and immune responses. These responses were induced by immunoglobulin G (IgG), hematopoietic cell kinase (HCK), major histocompatibility complex class II (MHC-II), lymphocyte-specific kinase (LCK), major histocompatibility complex class I (MHC-I), activator of transcription 1 (STAT1), interferon, B7-CD28, and tumor necrosis factor (TNF). Supplementary Material 6: source codes.
