# Supplementary material for: Identification of a Prognosis-Related Risk Signature for Bladder Cancer to Predict Survival and Immune Landscapes
Source: J Immunol Res. 2021 Oct 18;2021:3236384. doi: 10.1155/2021/3236384 (PMC8545590; doi:10.1155/2021/3236384)
Supplement: Supplementary Materials — Supplementary Figure 1: effects of the single factor on patient outcome in GSE13507. (A–E) The Kaplan–Meier survival curves for patients with bladder cancer in GSE13507, stratified according to the expression levels of APOL2, CLEC2D, GBP2, GSDMB, and TNFRSF14 (high vs. low); comparisons of the median survival time in both groups with log-rank tests (p = 0.049, p = 0.0056, p = 0.0018, p = 0.049, and p = 0.027, respectively). (F–J) ROC curve analysis of the prognostic accuracy of APOL2, CLEC2D, GBP2, GSDMB, and TNFRSF14 in GSE13507. Supplementary Figure 2: effects of the single factor on patient outcome in GSE32894. (A–E) The Kaplan–Meier survival curves for patients with BLCA in GSE32894, stratified according to the expression levels of APOL2, CLEC2D, GBP2, GSDMB, and TNFRSF14 (high vs. low); comparisons of the median survival time in both groups with log-rank tests (p < 0.0001, p = 0.00021, p = 0.00049, p = 0.00053, and p = 0.037, respectively). (F–J) ROC curve analysis of the prognostic accuracy of APOL2, CLEC2D, GBP2, GSDMB, and TNFRSF14 in GSE32894. Supplementary Figure 3: effects of the single factor on patient outcome in Mariathasan S's cohort. (A–E) The Kaplan–Meier survival curves for patients with BLCA in Mariathasan S's cohort, stratified according to the expression levels of APOL2, CLEC2D, GBP2, GSDMB, and TNFRSF14 (high vs. low); comparisons of the median survival time in both groups with log-rank tests (p = 0.018, p = 0.0018, p = 0.03, p = 0.0064, and p = 0.22, respectively). (F–J) ROC curve analysis of the prognostic accuracy of APOL2, CLEC2D, GBP2, GSDMB, and TNFRSF14 in Mariathasan S's cohort. Supplementary Figure 4: effects of the robust model on patient outcome in GSE13507. (A–F) The subgroups' Kaplan–Meier analysis of risk score. Supplementary Figure 5: tumor microenvironment analysis. (A–C) The difference of 22 kinds of immune cells between the high-expression group and the low-expression group according to the expression levels of CLEC2D and GBP2 [file 3236384.f1.docx]

Supplementary Figure 1.


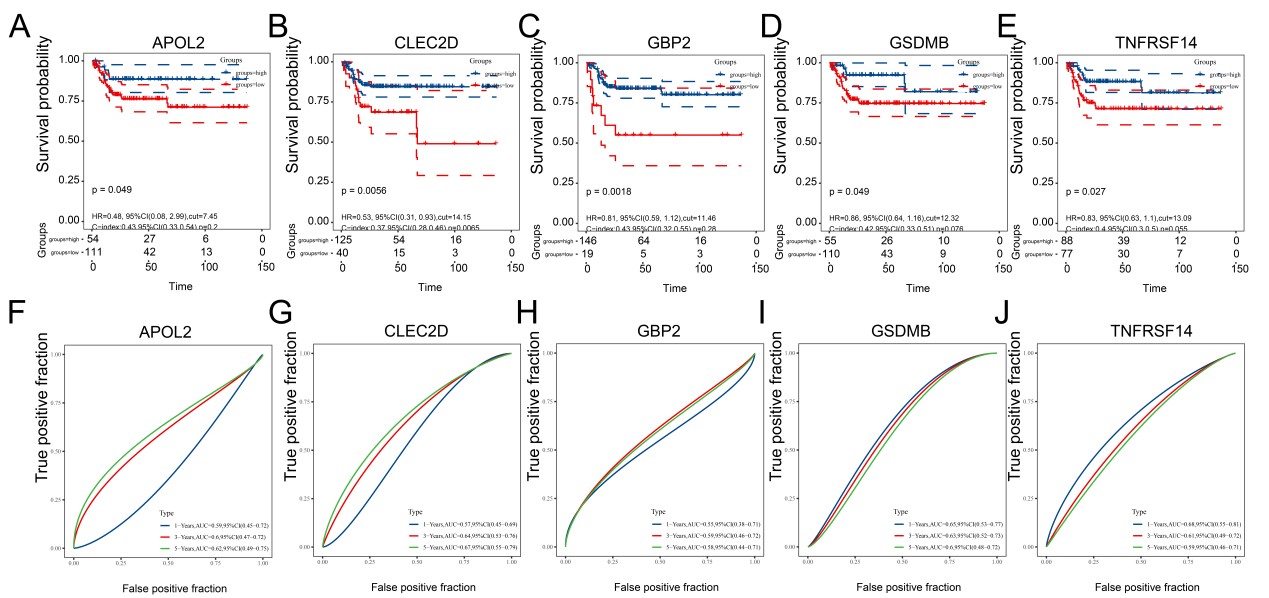


Supplementary Figure 2.


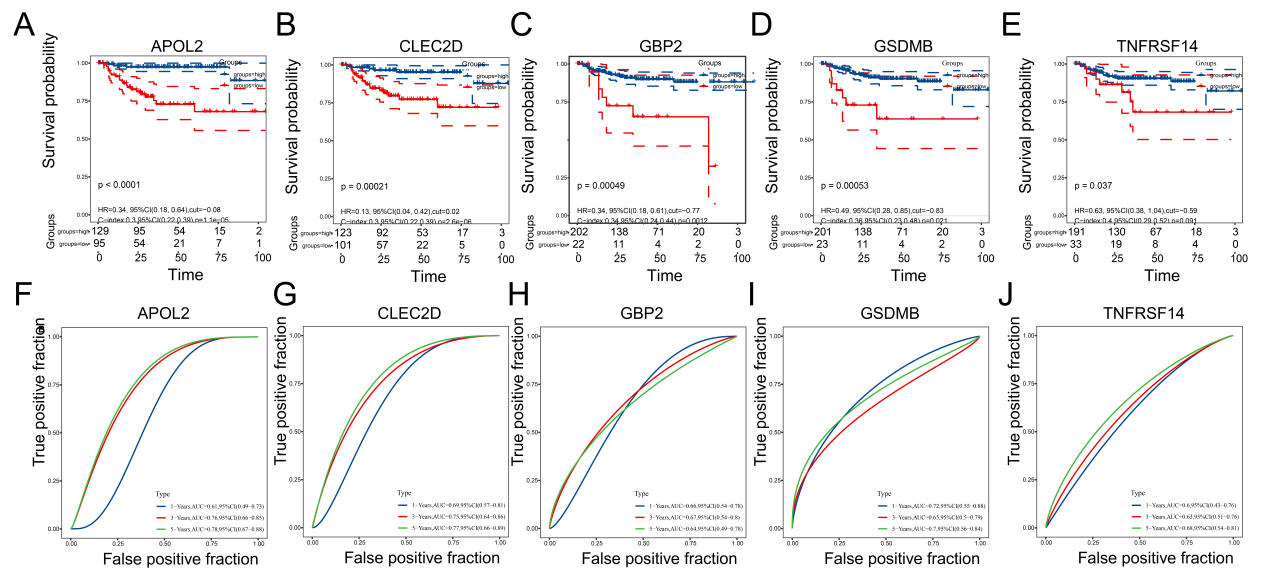


Supplementary Figure 3.


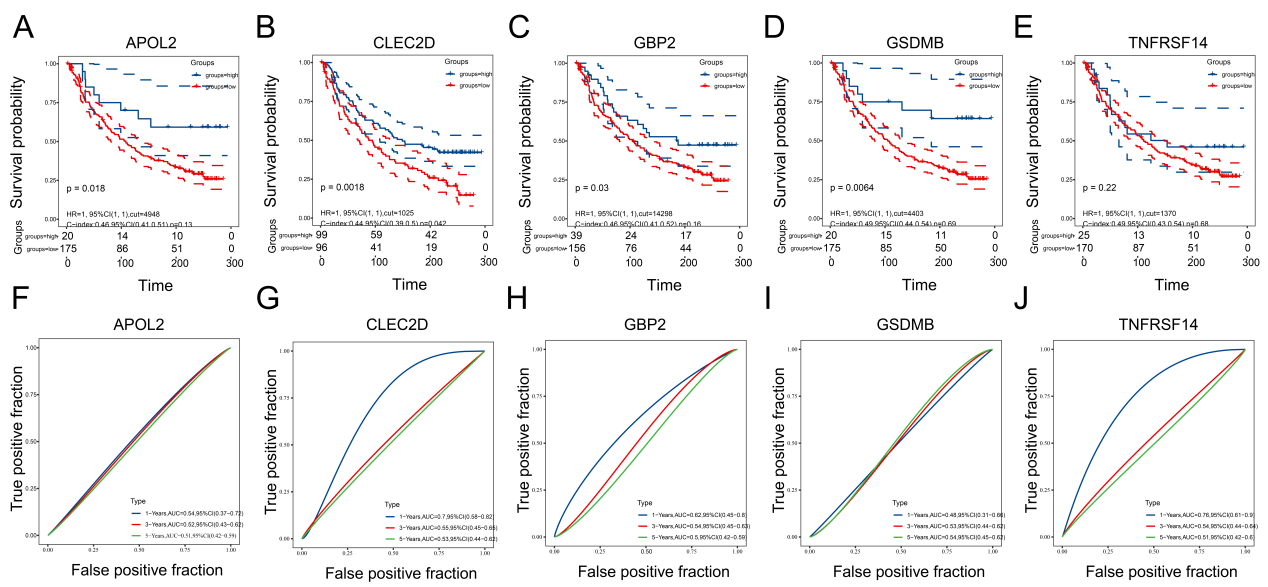


Supplementary Figure 4.


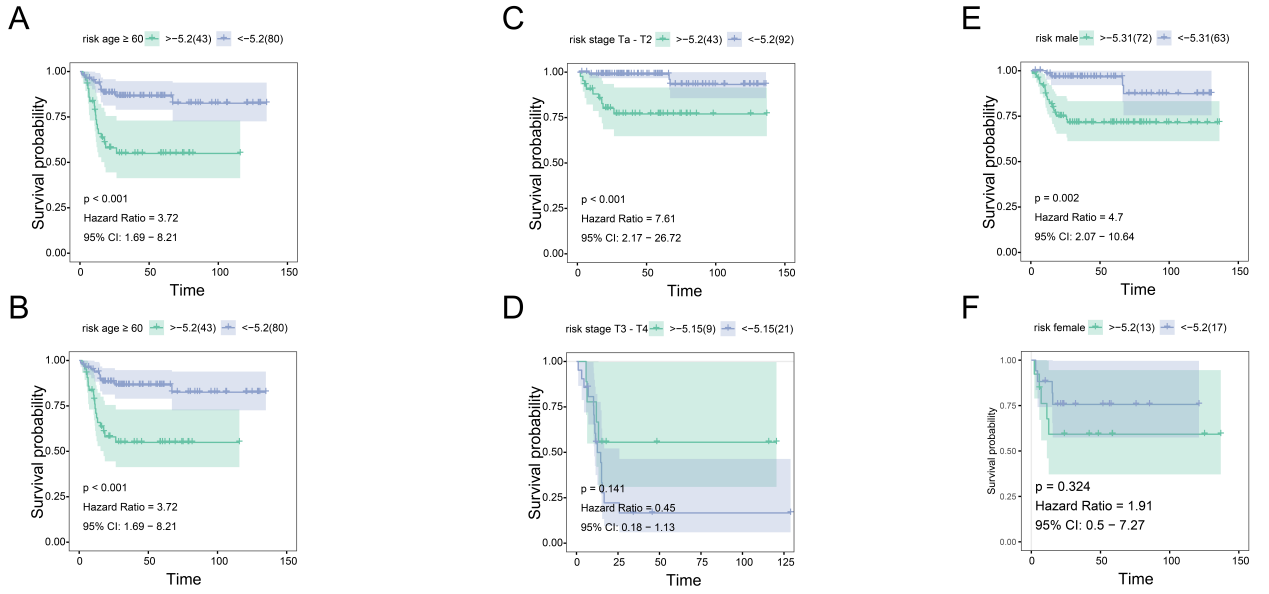


Supplementary Figure 5.


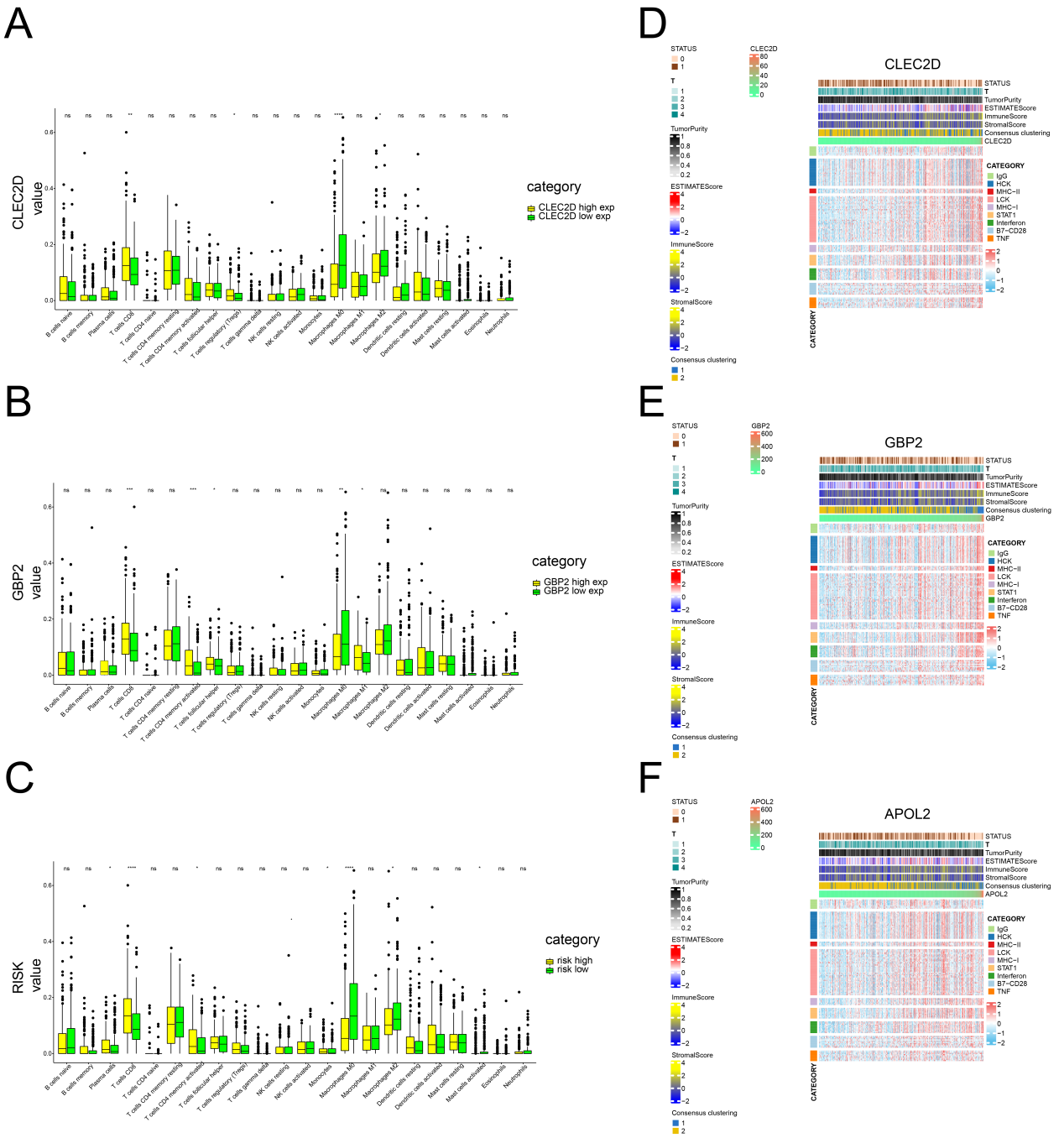


Supplementary source code

rbsurv

install.packages("BiocManager")

BiocManager::install("rbsurv", version = "3.9")

library(rbsurv)

setwd("C:/Users/Administrator/Desktop")

Mydata<-read.csv(file = "H:\\版本二\\R train input.csv",header=TRUE,row.names = 1)

##########################

#head(Mydata)

o=apply(Mydata,2,as.numeric)

row.names(o) <- row.names(Mydata)

o=o[c(1:424),c(1:346)]#######################################################################################################

#o=as.numeric(o)

Mydata[426, ]#############################################################################################################

time=Mydata[425,]#########################################################################################################

time=as.numeric(time)

time

status=Mydata[426,]########################################################################################################

status=as.numeric(status)

status

#time a vector for survival times

#status a vector for survival status, 0=censored, 1=event

#x a matrix for expression values (genes in rows, samples in columns)

#z a matrix for risk factors

#alpha a significance level for evaluating risk factors

#gene.ID a vector for gene IDs; if NULL, row numbers are assigned.

#method a character string specifying the method for tie handling.

#n.iter the number of iterations for gene selection

#n.fold the number of partitions of samples

#n.seq the number of sequential runs or multiple models

#seed a seed for sample partitioning

#max.n.genes the maximum number of genes considered M1....M30

#time <- Mydata$Time

#status <- Mydata$Status

#z <- cbind(Mydata$Age, Mydata$Gender)

#fit <- rbsurv(time=time, status=status, x=o, method="efron", max.n.genes=20, n.iter=100, n.seq=3,n.fold=3)

#fit <- rbsurv(time=time, status=status, x=o, method="efron", max.n.genes=100, n.iter=100, n.seq=3)

fit <- rbsurv(time=time, status=status, x=o, method="efron", max.n.genes=30, n.iter=100, n.seq=1,n.fold=3) ###################################

fit$model

#############

v1 <- unlist(fit)

v1

write.table (v1, file ="H:\\版本二\\trainset rbsurv output.csv", sep ="", row.names =TRUE, col.names =TRUE, quote =TRUE)###########################

#######################保存

#x

#file

#sep

#row.names

#col.names

#quote

GSEA

use strict;

use warnings;

my %hash=();

open(RF,"gene.txt") or die $!;

while(my $line=<RF>){

chomp($line);

$hash{$line}=1;

}

close(RF);

open(RF,"symbol.txt") or die $!;

open(WF,">m6Aexp.txt") or die $!;

while(my $line=<RF>){

if($.==1){

print WF $line;

next;

}

my @arr=split(/\t/,$line);

my @zeroArr=split(/\|/,$arr[0]);

if($zeroArr[0] eq "VIRMA"){

$line=~s/VIRMA/KIAA1429/g;

print WF $line;

delete($hash{"KIAA1429"});

}

elsif(exists $hash{$zeroArr[0]}){

print WF $line;

delete($hash{$zeroArr[0]});

}

}

close(WF);

close(RF);

foreach my $key(keys %hash){

print $key . "\n";

}

CIBERSORT

#install.packages('e1071')

#if (!requireNamespace("BiocManager", quietly = TRUE))

# install.packages("BiocManager")

#BiocManager::install("preprocessCore")

#if (!requireNamespace("BiocManager", quietly = TRUE))

# install.packages("BiocManager")

#BiocManager::install("limma")

library("limma")

setwd("H:\\16 膀胱癌\\1 原始数据\\1 tcga 433膀胱癌\\2 tcga 免疫浸润数据挖掘\\1 CIBERSORFT 免疫评分获取")

rt=read.table("symbol.txt",sep="\t",header=T,check.names=F)

rt=as.matrix(rt)

rownames(rt)=rt[,1]

exp=rt[,2:ncol(rt)]

dimnames=list(rownames(exp),colnames(exp))

data=matrix(as.numeric(as.matrix(exp)),nrow=nrow(exp),dimnames=dimnames)

data=avereps(data)

#group=sapply(strsplit(colnames(data),"\\-"),"[",4)

#group=sapply(strsplit(group,""),"[",1)

#group=gsub("2","1",group)

#data=data[,group==0]

data=data[rowMeans(data)>0,]

v <-voom(data, plot = F, save.plot = F)

out=v$E

out=rbind(ID=colnames(out),out)

write.table(out,file="uniq.symbol.txt",sep="\t",quote=F,col.names=F)

source("ssGSEA18.CIBERSORT.R")

results=CIBERSORT("ref.txt", "uniq.symbol.txt", perm=100, QN=TRUE)

Immune heatmap

#R.4.0.3

#library(devtools)

#install_github("jokergoo/ComplexHeatmap")

#install.packages("NMF")

#install.packages("ggplot")

#install.packages("devtools")

#install.packages("ggplot2")

#install.packages("gplots")

#install.packages("oompaBase")

#install.packages("Biobase")

# set working path

workdir <- "D:\\膀胱癌鲁棒性\\免疫\\heatmap"; setwd(workdir)

# customized function

standarize.fun <- function(indata=NULL, halfwidth=NULL, centerFlag=T, scaleFlag=T) {

outdata=t(scale(t(indata), center=centerFlag, scale=scaleFlag))

if (!is.null(halfwidth)) {

outdata[outdata>halfwidth]=halfwidth

outdata[outdata<(-halfwidth)]= -halfwidth

}

return(outdata)

}

# load R package

library(ComplexHeatmap)

library(gplots)

library(ggplot2)

library(RColorBrewer)

library(oompaBase)

# set colors

blue <- "#5bc0eb"

yellow <- "#fde74c"

green <- "#9bc53d"

red <- "#f25f5c"

purple <- "#531f7a"

grey <- "#8693ab"

orange <- "#fa7921"

white <- "#f2d7ee"

darkred <- "#F2042C"

lightred <- "#FF7FBF"

lightblue <- "#B2EBFF"

darkblue <- "#1d00ff"

cherry <- "#700353"

lightgrey <- "#dcddde"

nake <- "#F8C364"

gold <- "#ECE700"

cyan <- "#00B3D0"

sun <- "#E53435"

peach <- "#E43889"

violet <- "#89439B"

soil <- "#EC7D21"

lightgreen <- "#54B642"

darkblue <- "#21498D"

darkgreen <- "#009047"

brown <- "#874118"

seagreen <- "#008B8A"

jco <- c("#2874C5","#EABF00","#5FC1C2","#C6524A","#80A7DE")

mycol <- brewer.pal(n = 12, name = "Paired")

# load data

dat <- read.table("blca.txt",sep = "\t",row.names = NULL,check.names = F,stringsAsFactors = F,header = T)

dat <- dat[!duplicated(dat$ID),]

rownames(dat) <- dat$ID; dat <- dat[,-1]

sinfo <- dat[,1:8]

dat <- as.data.frame(t(dat[,setdiff(colnames(dat),colnames(sinfo))]))

anno <- read.table("annotation.txt",sep = "\t",row.names = NULL,check.names = F,stringsAsFactors = F,header = T)

anno <- anno[!duplicated(anno$GENE),]

rownames(anno) <- anno$GENE

comgene <- intersect(rownames(anno),rownames(dat))

anno <- anno[comgene,-1,drop = F]

dat <- dat[rownames(anno),]

# create annotation

annCol <- sinfo

annCol$StromalScore <- scale(annCol$StromalScore)

annCol$ImmuneScore <- scale(annCol$ImmuneScore)

annCol$ESTIMATEScore <- scale(annCol$ESTIMATEScore)

annCol <- annCol[order(annCol$Immunoprotease),]

annRow <- anno

annRow$CATEGORY <- factor(annRow$CATEGORY, levels = unique(annRow$CATEGORY))

annColors <- list("Consensus clustering" = c("1" = jco[1],"2" = jco[2]),

"T" = c("1" = alpha(seagreen,0.2),"2" = alpha(seagreen,0.4),"3" = alpha(seagreen,0.7),"4" = seagreen),

"STATUS" = c("0" = alpha(orange,0.3),"1" = brown),

"Immunoprotease" = NMF:::ccRamp(c("seagreen1","coral1"), 64),

"StromalScore" = blueyellow(64),

"ImmuneScore" = blueyellow(64),

"ESTIMATEScore" = bluered(64),

"TumorPurity" = NMF:::ccRamp(c("grey90","black"),64),

"CATEGORY" = c("B7-CD28" = mycol[1],"HCK" = mycol[2],

"IgG" = mycol[3], "Interferon" = mycol[4],

"LCK" = mycol[5], "MHC-II" = mycol[6],

"STAT1" = mycol[7], "TNF" = mycol[8],"MHC-I" = mycol[9]))

indata <- standarize.fun(log2(dat + 1), halfwidth = 2)

p <- pheatmap(indata[,rownames(annCol)],

cluster_rows = F,

cluster_cols = F,

show_rownames = F,

show_colnames = F,

#color = greenred(64),

color = NMF:::ccRamp(c("#5bc0eb", "white", "#f25f5c"),64),

border_color = NA,

annotation_col = annCol,

annotation_row = annRow,

annotation_colors = annColors,

cellwidth = 0.6,

cellheight = 2,

gaps_row = cumsum(table(annRow$CATEGORY)))

pdf("heatmap_blca.pdf", width = 10,height = 8)

draw(p, annotation_legend_side = "left")

invisible(dev.off())

Drug targets

workdir <- "G:/膀胱癌/YT/11"; setwd(workdir)

# customized functions

countToFpkm <- function(counts, effLen){

N <- sum(counts)

exp( log(counts) + log(1e9) - log(effLen) - log(N) )

}

fpkmToTpm <- function(fpkm)

{

exp(log(fpkm) - log(sum(fpkm)) + log(1e6))

}

display.progress = function (index, totalN, breakN=20) {

if ( index %% ceiling(totalN/breakN) ==0 ) {

cat(paste(round(index*100/totalN), "% ", sep=""))

}

}

# load R package

library(TCGAbiolinks)

library(impute)

library(pRRophetic)

library(SimDesign)

library(ggplot2)

library(cowplot)

library(patchwork)

# load annotation

Ginfo <- read.table("overlapTable27.txt",sep = "\t",row.names = 1,check.names = F,stringsAsFactors = F,header = T)

Ginfo <- Ginfo[which(Ginfo$genetype == "protein_coding"),]

# download prostate cancer expression

expquery <- GDCquery(project = "TCGA-BLCA",

data.category = "Transcriptome Profiling",

data.type = "Gene Expression Quantification",

workflow.type = "HTSeq - Counts"

)

GDCdownload(expquery,directory = "GDCdata")

expquery2 <- GDCprepare(expquery,directory = "GDCdata",summarizedExperiment = T)

expMatrix <- TCGAanalyze_Preprocessing(expquery2)

colnames(expMatrix) <- substr(colnames(expMatrix), start = 1,stop = 16)

normsamples <- colnames(expMatrix)[which(substr(colnames(expMatrix),14,16) == "11A")] # get normal samples

tumorsamples <- colnames(expMatrix)[which(substr(colnames(expMatrix),14,16) == "01A")] # get tumor samples

expMatrix <- expMatrix[,tumorsamples]; expMatrix <- as.data.frame(expMatrix[rowSums(expMatrix) > 0,])

comgene <- intersect(rownames(expMatrix),rownames(Ginfo))

count <- as.data.frame(expMatrix)[comgene,]; Ginfo <- Ginfo[comgene,]

count$gene <- Ginfo$genename; count <- count[!duplicated(count$gene),]; Ginfo <- Ginfo[rownames(count),]; rownames(count) <- count$gene; count <- count[,-ncol(count)]

write.table(count,"TCGA_BLCA_Count.txt", quote=F, row.names=T,col.names = NA,sep = "\t")

fpkms <- apply(count, 2, countToFpkm, effLen = Ginfo$unqlen)

tpms <- as.data.frame(round(apply(fpkms,2,fpkmToTpm),2))

write.table(tpms, "TCGA_BLCA_TPMS.txt", quote=F, row.names=T,col.names = NA,sep = "\t")

auc <- read.table("CTRP_AUC_raw.txt",sep = "\t",row.names = NULL,check.names = F,stringsAsFactors = F,header = T) # Supplementary Data Set 3

auc$comb <- paste(auc$master_cpd_id,auc$master_ccl_id,sep = "-")

auc <- apply(auc[,"area_under_curve",drop = F], 2, function(x) tapply(x, INDEX=factor(auc$comb), FUN=max, na.rm=TRUE))

auc <- as.data.frame(auc)

auc$master_cpd_id <- sapply(strsplit(rownames(auc),"-",fixed = T),"[",1)

auc$master_ccl_id <- sapply(strsplit(rownames(auc),"-",fixed = T),"[",2)

auc <- reshape(auc,

direction = "wide",

timevar = "master_cpd_id",

idvar = "master_ccl_id")

colnames(auc) <- gsub("area_under_curve.","",colnames(auc),fixed = T)

ctrp.ccl.anno <- read.table("CTRP_ccl_anno.txt",sep = "\t",row.names = NULL,check.names = F,stringsAsFactors = F,header = T) # Supplementary Data Set 1

ctrp.cpd.anno <- read.delim("CTRP_cpd_anno.txt",sep = "\t",row.names = NULL,check.names = F,stringsAsFactors = F,header = T) # Supplementary Data Set 2

write.table(auc,"CTRP_AUC.txt",sep = "\t",row.names = F,col.names = T,quote = F)

ctrp.auc <- read.table("CTRP_AUC.txt",sep = "\t",row.names = 1,check.names = F,stringsAsFactors = F,header = T)

prism.auc <- read.delim("PRISM_AUC.txt",sep = "\t",row.names = 1,check.names = F,stringsAsFactors = F,header = T)

prism.ccl.anno <- prism.auc[,1:5]

prism.auc <- prism.auc[,-c(1:5)]

ctrp.auc <- ctrp.auc[,apply(ctrp.auc,2,function(x) sum(is.na(x))) < 0.2*nrow(ctrp.auc)]

prism.auc <- prism.auc[,apply(prism.auc,2,function(x) sum(is.na(x))) < 0.2*nrow(prism.auc)]

rmccl <- paste0("CCL",na.omit(ctrp.ccl.anno[which(ctrp.ccl.anno$ccle_primary_site == "haematopoietic_and_lymphoid_tissue"),"master_ccl_id"]))

rownames(ctrp.auc) <- paste0("CCL",rownames(ctrp.auc))

ctrp.auc <- ctrp.auc[setdiff(rownames(ctrp.auc),rmccl),]

ctrp.auc.knn <- impute.knn(as.matrix(ctrp.auc))$data

prism.auc.knn <- impute.knn(as.matrix(prism.auc))$data

ctrp.auc.knn <- ctrp.auc.knn/ceiling(max(ctrp.auc.knn))

prism.auc.knn <- prism.auc.knn/ceiling(max(prism.auc.knn))

ccl.expr <- read.table("CCLE_RNAseq_rsem_genes_tpm_20180929.txt.gz",sep = "\t",row.names = 1,check.names = F,stringsAsFactors = F,header = T)

ccl.expr <- ccl.expr[,-1]; rownames(ccl.expr) <- sapply(strsplit(rownames(ccl.expr),".",fixed = T),"[",1)

comgene <- intersect(rownames(ccl.expr),rownames(Ginfo))

ccl.expr <- ccl.expr[comgene,]

ccl.expr$gene <- Ginfo[comgene,"genename"]; ccl.expr <- ccl.expr[!duplicated(ccl.expr$gene),]; rownames(ccl.expr) <- ccl.expr$gene; ccl.expr <- ccl.expr[,-ncol(ccl.expr)]

## CTRP

trainExpr <- log2(ccl.expr + 1)

colnames(trainExpr) <- sapply(strsplit(colnames(trainExpr),"_",fixed = T),"[",1)

trainPtype <- as.data.frame(ctrp.auc.knn)

ccl.name <- ccl.miss <- c()

for (i in rownames(trainPtype)) {

if(!is.element(gsub("CCL","",i),ctrp.ccl.anno$master_ccl_id)) {

cat(i,"\n")

ccl.miss <- c(ccl.miss, i)

ccl.name <- c(ccl.name, i)

} else {

ccl.name <- c(ccl.name, ctrp.ccl.anno[which(ctrp.ccl.anno$master_ccl_id == gsub("CCL","",i)),"ccl_name"])

}

}

cpd.name <- cpd.miss <- c()

for (i in colnames(trainPtype)) {

if(!is.element(i,ctrp.cpd.anno$master_cpd_id)) {

cat(i,"\n")

cpd.miss <- c(cpd.miss, i)

cpd.name <- c(cpd.name, i)

} else {

cpd.name <- c(cpd.name, ctrp.cpd.anno[which(ctrp.cpd.anno$master_cpd_id == i),"cpd_name"])

}

}

rownames(trainPtype) <- ccl.name

trainPtype <- trainPtype[setdiff(rownames(trainPtype),ccl.miss),]

colnames(trainPtype) <- cpd.name

trainPtype <- trainPtype[,setdiff(colnames(trainPtype),cpd.miss)]

comccl <- intersect(rownames(trainPtype),colnames(trainExpr))

trainExpr <- trainExpr[,comccl]

trainPtype <- trainPtype[comccl,]

testExpr <- log2(tpms + 1)

comgene <- intersect(rownames(trainExpr),rownames(testExpr))

trainExpr <- as.matrix(trainExpr[comgene,])

testExpr <- as.matrix(testExpr[comgene,])

outTab <- NULL

for (i in 1:ncol(trainPtype)) {

display.progress(index = i,totalN = ncol(trainPtype))

d <- colnames(trainPtype)[i]

tmp <- log2(as.vector(trainPtype[,d]) + 0.00001)

ptypeOut <- quiet(calcPhenotype(trainingExprData = trainExpr,

trainingPtype = tmp,

testExprData = testExpr,

powerTransformPhenotype = F,

selection = 1))

ptypeOut <- 2^ptypeOut - 0.00001

outTab <- rbind.data.frame(outTab,ptypeOut)

}

dimnames(outTab) <- list(colnames(trainPtype),colnames(testExpr))

ctrp.pred.auc <- outTab

write.table(ctrp.pred.auc,"ctrp.pred.auc.txt",sep = "\t",row.names = T,col.names = NA,quote = F)

## PRISM

trainExpr <- log2(ccl.expr + 1)

colnames(trainExpr) <- sapply(strsplit(colnames(trainExpr),"_",fixed = T),"[",1)

trainPtype <- as.data.frame(prism.auc.knn)

rownames(trainPtype) <- prism.ccl.anno[rownames(trainPtype),"cell_line_display_name"]

#colnames(trainPtype) <- sapply(strsplit(colnames(trainPtype)," (",fixed = T), "[",1)

comccl <- intersect(rownames(trainPtype),colnames(trainExpr))

trainExpr <- trainExpr[,comccl]

trainPtype <- trainPtype[comccl,]

testExpr <- log2(tpms + 1)

comgene <- intersect(rownames(trainExpr),rownames(testExpr))

trainExpr <- as.matrix(trainExpr[comgene,])

testExpr <- as.matrix(testExpr[comgene,])

outTab <- NULL

for (i in 1:ncol(trainPtype)) {

display.progress(index = i,totalN = ncol(trainPtype))

d <- colnames(trainPtype)[i]

tmp <- log2(as.vector(trainPtype[,d]) + 0.00001)

ptypeOut <- quiet(calcPhenotype(trainingExprData = trainExpr,

trainingPtype = tmp,

testExprData = testExpr,

powerTransformPhenotype = F,

selection = 1))

ptypeOut <- 2^ptypeOut - 0.00001

outTab <- rbind.data.frame(outTab,ptypeOut)

}

dimnames(outTab) <- list(colnames(trainPtype),colnames(testExpr))

prism.pred.auc <- outTab

write.table(prism.pred.auc,"prism.pred.auc.txt",sep = "\t",row.names = T,col.names = NA,quote = F)

# identical potential drug target

Sinfo <- data.frame(samID = colnames(tpms),

TNFRSF14 = log2(as.numeric(tpms["TNFRSF14",]) + 1),

row.names = colnames(tpms),

stringsAsFactors = F)

top.sam <- Sinfo[Sinfo$TNFRSF14 >= quantile(Sinfo$TNFRSF14,probs = seq(0,1,0.1))[10],]

bot.sam <- Sinfo[Sinfo$TNFRSF14 <= quantile(Sinfo$TNFRSF14,probs = seq(0,1,0.1))[2],]

ctrp.log2fc <- c()

for (i in 1:nrow(ctrp.pred.auc)) {

display.progress(index = i,totalN = nrow(ctrp.pred.auc))

d <- rownames(ctrp.pred.auc)[i]

a <- mean(as.numeric(ctrp.pred.auc[d,rownames(top.sam)]))

b <- mean(as.numeric(ctrp.pred.auc[d,rownames(bot.sam)]))

fc <- b/a

log2fc <- log2(fc); names(log2fc) <- d

ctrp.log2fc <- c(ctrp.log2fc,log2fc)

}

candidate.ctrp <- ctrp.log2fc[ctrp.log2fc < -0.1]

prism.log2fc <- c()

for (i in 1:nrow(prism.pred.auc)) {

display.progress(index = i,totalN = nrow(prism.pred.auc))

d <- rownames(prism.pred.auc)[i]

a <- mean(as.numeric(prism.pred.auc[d,rownames(top.sam)]))

b <- mean(as.numeric(prism.pred.auc[d,rownames(bot.sam)]))

fc <- b/a

log2fc <- log2(fc); names(log2fc) <- d

prism.log2fc <- c(prism.log2fc,log2fc)

}

candidate.prism <- prism.log2fc[prism.log2fc < -0.1]

ctrp.cor <- ctrp.cor.p <- c()

for (i in 1:nrow(ctrp.pred.auc)) {

display.progress(index = i,totalN = nrow(ctrp.pred.auc))

d <- rownames(ctrp.pred.auc)[i]

a <- as.numeric(ctrp.pred.auc[d,rownames(Sinfo)])

b <- as.numeric(Sinfo$TNFRSF14)

r <- cor.test(a,b,method = "spearman")$estimate; names(r) <- d

p <- cor.test(a,b,method = "spearman")$p.value; names(p) <- d

ctrp.cor <- c(ctrp.cor,r)

ctrp.cor.p <- c(ctrp.cor.p,p)

}

candidate.ctrp2 <- ctrp.cor[ctrp.cor > 0.2]

ctrp.candidate <- intersect(names(candidate.ctrp),names(candidate.ctrp2))

prism.cor <- prism.cor.p <- c()

for (i in 1:nrow(prism.pred.auc)) {

display.progress(index = i,totalN = nrow(prism.pred.auc))

d <- rownames(prism.pred.auc)[i]

a <- as.numeric(prism.pred.auc[d,rownames(Sinfo)])

b <- as.numeric(Sinfo$TNFRSF14)

r <- cor.test(a,b,method = "spearman")$estimate; names(r) <- d

p <- cor.test(a,b,method = "spearman")$p.value; names(p) <- d

prism.cor <- c(prism.cor,r)

prism.cor.p <- c(prism.cor.p,p)

}

candidate.prism2 <- prism.cor[prism.cor > 0.3]

prism.candidate <- intersect(names(candidate.prism),names(candidate.prism2))

# generate plot

darkblue <- "#0772B9"

lightblue <- "#48C8EF"

cor.data <- data.frame(drug = ctrp.candidate,

r = ctrp.cor[ctrp.candidate],

p = -log10(ctrp.cor.p[ctrp.candidate]))

p1 <- ggplot(data = cor.data,aes(r,forcats::fct_reorder(drug,r,.desc = T))) +

geom_segment(aes(xend=0,yend=drug),linetype = 2) +

geom_point(aes(size=p),col = darkblue) +

scale_size_continuous(range =c(2,8)) +

scale_x_continuous(breaks = c(0, 0.2, 0.4,0.6,0.8),

expand = expansion(mult = c(0.01,.1))) +

theme_classic() +

labs(x = "Correlation coefficient", y = "", size = bquote("-log"[10]~"("~italic(P)~"-value)")) +

theme(legend.position = "bottom",

axis.line.y = element_blank())

p1

cor.data <- data.frame(drug = prism.candidate,

r = prism.cor[prism.candidate],

p = -log10(prism.cor.p[prism.candidate]))

cor.data$drug <- sapply(strsplit(cor.data$drug," (",fixed = T), "[",1)

p2 <- ggplot(data = cor.data,aes(r,forcats::fct_reorder(drug,r,.desc = T))) +

geom_segment(aes(xend=0,yend=drug),linetype = 2) +

geom_point(aes(size=p),col = darkblue) +

scale_size_continuous(range =c(2,8)) +

scale_x_continuous(breaks = c(0, 0.2,0.4,0.6,0.8),

expand = expansion(mult = c(0.01,.1))) +

theme_classic() +

labs(x = "Correlation coefficient", y = "", size = bquote("-log"[10]~"("~italic(P)~"-value)")) +

theme(legend.position = "bottom",

axis.line.y = element_blank())

p2

ctrp.boxdata <- NULL

for (d in ctrp.candidate) {

a <- as.numeric(ctrp.pred.auc[d,rownames(top.sam)])

b <- as.numeric(ctrp.pred.auc[d,rownames(bot.sam)])

p <- wilcox.test(a,b)$p.value

s <- as.character(cut(p,c(0,0.001,0.01,0.05,1),labels = c("***","**","*","")))

ctrp.boxdata <- rbind.data.frame(ctrp.boxdata,

data.frame(drug = d,

auc = c(a,b),

p = p,

s = s,

group = rep(c("High TNFRSF14","Low TNFRSF14"),c(nrow(top.sam),nrow(bot.sam))),

stringsAsFactors = F),

stringsAsFactors = F)

}

p3 <- ggplot(ctrp.boxdata, aes(drug, auc, fill=group)) +

geom_boxplot(aes(col = group),outlier.shape = NA) +

# geom_text(aes(drug, y=min(auc) * 1.1,

# label=paste("p=",formatC(p,format = "e",digits = 1))),

# data=ctrp.boxdata,

# inherit.aes=F) +

geom_text(aes(drug, y=max(auc)),

label=ctrp.boxdata$s,

data=ctrp.boxdata,

inherit.aes=F) +

scale_fill_manual(values = c(darkblue, lightblue)) +

scale_color_manual(values = c(darkblue, lightblue)) +

xlab(NULL) + ylab("Estimated AUC value") +

theme_classic() +

theme(axis.text.x = element_text(angle = 45, hjust = 0.5,vjust = 0.5,size = 10),

legend.position = "bottom",

legend.title = element_blank())

dat <- ggplot_build(p3)$data[[1]]

p3 <- p3 + geom_segment(data=dat, aes(x=xmin, xend=xmax, y=middle, yend=middle), color="white", inherit.aes = F)

prism.boxdata <- NULL

for (d in prism.candidate) {

a <- as.numeric(prism.pred.auc[d,rownames(top.sam)])

b <- as.numeric(prism.pred.auc[d,rownames(bot.sam)])

p <- wilcox.test(a,b)$p.value

s <- as.character(cut(p,c(0,0.001,0.01,0.05,1),labels = c("***","**","*","")))

prism.boxdata <- rbind.data.frame(prism.boxdata,

data.frame(drug = d,

auc = c(a,b),

p = p,

s = s,

group = rep(c("High TNFRSF14","Low TNFRSF14"),c(nrow(top.sam),nrow(bot.sam))),

stringsAsFactors = F),

stringsAsFactors = F)

}

prism.boxdata$drug <- sapply(strsplit(prism.boxdata$drug," (",fixed = T), "[",1)

p4 <- ggplot(prism.boxdata, aes(drug, auc, fill=group)) +

geom_boxplot(aes(col = group),outlier.shape = NA) +

# geom_text(aes(drug, y=min(auc) * 1.1,

# label=paste("p=",formatC(p,format = "e",digits = 1))),

# data=prism.boxdata,

# inherit.aes=F) +

geom_text(aes(drug, y=max(auc)),

label=prism.boxdata$s,

data=prism.boxdata,

inherit.aes=F) +

scale_fill_manual(values = c(darkblue, lightblue)) +

scale_color_manual(values = c(darkblue, lightblue)) +

xlab(NULL) + ylab("Estimated AUC value") +

theme_classic() +

theme(axis.text.x = element_text(angle = 45, hjust = 0.5,vjust = 0.5,size = 10),

legend.position = "bottom",

legend.title = element_blank())

dat <- ggplot_build(p4)$data[[1]]

p4 <- p4 + geom_segment(data=dat, aes(x=xmin, xend=xmax, y=middle, yend=middle), color="white", inherit.aes = F)

plot_grid(p1, p3, p2, p4, labels=c("A", "", "B", ""),

ncol=2,

rel_widths = c(2, 2))

ggsave(filename = "drug target.pdf",width = 10,height = 10)

save.image("YT.RData")
